# Mechanical and Processing Properties of Plasticised PVC/Wood Composites

**DOI:** 10.3390/polym16152204

**Published:** 2024-08-02

**Authors:** Krzysztof Lewandowski, Piotr Altmajer, Zuzanna Borkowska, Katarzyna Skórczewska

**Affiliations:** Department of Polymer Technology and Protective Coatings, Faculty of Chemical Technology and Engineering, Bydgoszcz University of Science and Technology, Seminaryjna 3, 85-326 Bydgoszcz, Poland; pioalt000@pbs.edu.pl (P.A.); zuzbor000@pbs.edu.pl (Z.B.)

**Keywords:** wood–polymer composite, soft PVC, flexible composites

## Abstract

The paper presents the results of testing the properties of wood–polymer composites (WPC) based on plasticised poly(vinyl chloride) (PVC-P). Materials with variable contents of wood filler (Arbocel C 320) or plasticiser (di-isononyl phthalate) were produced and then analysed. The share of wood flour in the material was up to 50 phr, and the plasticiser content was up to 40 phr. Functional properties, such as tensile properties, mechanical properties at variable temperature (DMTA), and water absorption, as well as processing properties such as rheological properties and analysis of the fusion process, were analysed. The influences of wood flour and plasticiser on the composites’ properties in the solid and melted state were found. For example, with 40 phr of plasticiser, increasing the filler share from 0 phr to 50 phr resulted in an increased tensile modulus from 18 MPa to 274 MPa and viscosity at a share rate of 20 s^−1^, from 721 Pa·s to 1581 Pa·s. However, increasing the share of plasticiser from 20 phr to 40 phr with 30 phr of filler reduces the value of these properties from 1760 MPa to 112 MPa and from 2768 Pa·s to 1151 Pa·s, respectively. It was also found that increasing the share of wood flour in the composite noticeably reduces the effectiveness of the plasticiser.

## 1. Introduction

The production of composites involves the physical modification of polymers to obtain materials with new properties [1]. It is interesting to use crushed plant parts as fillers. In this case, wood–polymer composites (WPCs) are obtained. WPCs have found wide application as a construction material. The components produced from WPCs can be used as a substitute for wood. Their advantage over wood is their high resistance to atmospheric properties of rotting and decay [2,3]. Currently, the global WPC market is USD 7.5 billion and is estimated to grow to USD 11.9 billion by 2028 [4].

Filler particle sizes can range from a few micrometres to a few millimetres [3,5]. The wood filler consists mainly of cellulose (45–50 wt.%), hemicellulose (20–25 wt.%), and lignin (20–30 wt.%) and has an extensive cellular structure that is typical of wood [6]. Due to the thermal stability of wood, it is important that composites made from it can be processed at temperatures below 195 °C [7]. For this reason, the possibilities of using thermoplastics as a polymer matrix for WPCs are limited. Thermoplastics such as polypropylene (PP), polyethylene (PE), or poly(vinyl chloride) PVCs are most commonly used as matrix [8,9,10,11], but also biodegradable polymers such as poly(lactic acid) PLA, poly(butylene succinate) PBS, and poly(butylene adipate-co-butylene terephthalate) PBAT [12,13,14,15]. The addition of wood filler primarily increases stiffness but can, nevertheless, reduce tensile strength and impact strength [16,17,18,19]. The addition of the filler also affects significant changes in processing properties, resulting in a significant increase in viscosity [20,21,22].

PVC is the second most frequently used polymer material for the production of WPC, after PE [19]. The result is due to the characteristics of PVC properties. It is a polymer with very high resistance to weather conditions, good stiffness, mechanical strength, and a low price [23]. WPC manufactured on the basis of unplasticised PVC is mainly used as terrace boards, extruded construction profiles, and siding [3].

PVC is a material that is very susceptible to modification. One common modified PVC variety is plasticised poly(vinyl chloride) (PVC-P). Thanks to the use of plasticisers, materials with increased flexibility and a reduced glass transition temperature are obtained. Such materials are used as, among other things, cable insulation, flexible hoses, gaskets, and toys [24]. Mainly phthalic acid esters, terephthalic acid esters, and linear dicarboxylic acid esters, but also epoxidised vegetable oils, e.g., epoxidised soybean oil, are used as plasticisers [24,25,26]. Phthalates are of particular importance in PVC-P technology; however, it should be noted that low-molecular-weight phthalates are subject to restrictions on use [24,27]. A representative of the phthalate plasticisers approved for use is the di-isononyl phthalate (DINP), which is characterised by very good plasticisation efficiency and low migration. Materials using it are widely used in external and internal applications that are particularly exposed to relatively high temperatures and require greater resistance to degradation, e.g., cable insulation, floor coverings, resistant flexible hoses, and extruded profiles [24,25,26,27].

It is also possible to develop WPCs characterised by increased flexibility. Their use is not as widespread compared to rigid WPC varieties. This may be indirectly related to the decidedly lower number of scientific reports on these varieties of composites. It has been shown that it is possible to obtain wood-filled composites on a plasticised poly(vinyl chloride) matrix. Separate research papers have investigated the effect of wood flour or plasticiser on the properties of WPC. It has been shown that an increase in stiffness, absorbability or density is to be expected with the growth of wood content [28]. On the contrary, the addition of a plasticiser will have the effect of reducing stiffness and improving impact strength [29,30]. In contrast, the paper presents the possibility of producing plastisols of plasticised emulsion poly(vinyl chloride) as a potential coating material [31].

The simultaneous modification of PVC with wood flour and plasticiser results in a composite system with complex interactions and interesting properties. These materials can be used as elements of flooring and wall-coverings systems and as extruded profiles for decorative and finishing elements complementing the range made of hard WPC varieties.

There is great interest in WPCs based on unplasticised PVC, but there is a lack of structured knowledge in area of PVC-P/WF composites. It is therefore worth conducting further research into the possibility of obtaining properties and the subsequent modification of composites based on wood filler and plasticised PVC.

The aim of the presented research work was to assess the influence of the components of wood–polymer composites based on a suspension poly(vinyl chloride) matrix with a plasticiser and wood flour. The analysis was performed for varying proportions of wood flour and plasticiser. Mechanical property tests were carried out for the composites produced. An analysis of processing properties, including as plasticisation tests and rheological tests, and an analysis of the change in elastic properties as a function of temperature (DMTA), not yet described in the literature, were also carried out.

## 2. Materials and Methods

### 2.1. Materials

Suspension poly(vinyl chloride) Neralit 601 (Spolana s. r. o., Neratovice, Czech Republic) with a K = 60 was used as the polymer material. PVC additives were also used: the heat stabiliser octyl tin mercaptide Patstab 2301 (Patcham, Holten, The Netherlands) and the lubricant paraffin wax Naftolube FTP (Chemson, Arnoldstein, Austria). Di-isononyl phthalate (DINP) (Brenntag Polska sp. z o. o., Kędzierzyn-Koźle, Poland) was used as the plasticiser. Arbocel C 320 wood flour (J. Rettenmaier & Söhne Gmbh + Co., Rosenberg, Germany) was used as the filler. Arbocel C 320 is a softwood product with a cubic structure. The main particle range is 200–500 µm.

### 2.2. Materials Processing and Preparation of Sample for Testing

The composition of the composites produced, together with their designation, is shown in Table 1. The selection of dry-blend ingredients was based on the technological practice of PVC processing. The thermal stabiliser (Patstab 2301) prevents polymer degradation and is an essential ingredient in PVC processing. Additional lubricant (Naftolube FTP) is added to facilitate processing, but its amount is very small so as not to significantly affect the properties of the manufactured materials [32,33]. The effect of wood flour on PVC-P was tested with a DINP content of 40 phr. With this share, a significant reduction in the glass transition temperature of PVC is observed, and the content is similar to the plasticiser content in materials intended for cable insulation, flexible hoses, and gaskets [25,33]. The influence of the plasticiser share on the properties of WPC was analysed for materials with a WF content of 30 phr. The lower range of plasticiser use was estimated in such a way as to obtain materials with a glass transition temperature close to 23 °C [34].

In the first step of processing, dry blends consisting of PVC, a thermal stabiliser, and a plasticiser were produced using a mixer (Battaggion, Bergamo, Italy) with two z-shaped mixing paddles rotating at speeds 16 min^−1^ and 32 min^−1^. The blending process was carried out at 90 °C. The preheating time for PVC with heat stabiliser and wax was 5 min, after which the plasticiser was added and mixed for a further 15 min. The mixture thus prepared was transferred to steel trays, cooled at 23 °C with gentle stirring for 10 min, and then transferred to sealed bags and left for a minimum of 24 h for conditioning. The dry blends thus prepared were intended for the manufacture of WPCs.

The wood flour was dried for a minimum of 3 h at 100 °C in a forced-air laboratory dryer. It was then mixed with dry blend in a separate container, using a bucket mixer, in the appropriate proportions (Table 1). Mixtures were dosing by the feeder to the main feed zone of the extruder. The extrusion output was set at 3 kg/h. A EPH 2 × 24 twin-screw extruder (Zamak Mercator, Skawina, Poland) was used for extrusion, with screw diameters of 2 × 24 mm at L/D = 40. The extruder was equipped with nine temperature zones (z), a head zone, and a connector. The temperature settings for each zone are summarised in Table 2. The rotation speed of the augers was set at 50 min^−1^ in co-rotating mode. Extrusion was carried out through a cylindrical head, and the extrudate was cooled in air, using a series of fans, and cut with a granulator to form pellets.

The fittings for the mechanical properties’ tests were manufactured by injection moulding. An Victory 120 injection moulding machine (Engel, Schwertberg, Austria) was used for this purpose. The process was carried out with the following parameters: dispensing speed, 0.2 m/s; specific back pressure, 100 bar; injection rate, 20 cm^3^/min s; cooling time, 10 s; clamping pressure, 60 MPa; clamping time, 20 s; and screw diameter, 40 mm. The temperature conditions of the individual zones are summarised in Table 2.

Plates measuring 100 mm × 100 mm × 2 mm were also shaped from the granules produced, using the pressing method. The processing temperature was 170 °C. The pre-heating time was 3 min, and the pressing under pressure (20 MPa) was 2 min. Strips 10 mm wide were cut from the plates, using punches, and samples were then cut from the strips for DMTA testing.

The materials, before processing by injection moulding or pressing method, were dried in a laboratory dryer for 4 h at 80 °C.

### 2.3. Testing Methods

#### 2.3.1. Determination of Tensile Properties

The test was carried out using a Zwick/Roell testing machine with a load cell with a nominal force of 5 kN, in accordance with EN ISO 527 [35]. Standardised type 1A samples were tested. The pretension was 0.1 MPa, the tensile modulus speed was 1 mm/min, and the test speed was 50 mm/min. The following were determined from the recorded force–strain relationships: elastic modulus, maximum stress, strain at maximum stress, stress at break, and strain at break. The measurement was performed in at least 5 repetitions for each material. Results are presented as mean value with standard deviation.

#### 2.3.2. Determination of the Glass Transition Temperature by DMTA

The determination of the glass transition temperature (*T_g_*) was performed using the dynamic thermomechanical analysis (DMTA) technique. The samples were in the shape of a rectangular measuring 10 mm × 30 mm × 2 mm. The test was carried out in a three-point bending system, with a support spacing of 20 mm, using a strain of 10 micrometres at an amplitude of 1 Hz. The measurement was carried out in the temperature range from −120 °C to 120 °C, using a heating rate of 3 °C/min. The changes in storage modulus, *E*′, and loss factor, tan*δ*, were recorded. The glass transition temperature was analysed based on the temperature dependence of *E*′ by determining the onset, inflection, and end point. The measurement was performed in at least 3 repetitions for each material. Results are presented as mean value with standard deviation.

#### 2.3.3. Determination of Water Absorption

The water absorption test was performed by immersing the previously weighted injection-mould samples in distilled water at 22 ± 2 °C and then re-weighting them after a specified time. The initial mass was considered to be materials containing the residual humidity obtained after conditioning for 7 days at a temperature of 23 °C and a humidity of approximately 50 ± 5%. The result is given as a percentage of the absorbed water related to the initial weight of the sample. Three measurements were taken for each material, from which the mean value was determined, together with the standard deviation. Measurements were taken after 24 h, 72 h, 168 h, 336 h, 504 h, 672 h, and 840 h.

#### 2.3.4. Determination of Rheological Properties

Rheological tests were performed using a LCR 7001 (Dynisco, Franklin, MA, USA) capillary rheometer. The measured temperature was 170 °C. Measurements were taken using cylindrical nozzles with a channel diameter of 2 mm, length of 30 mm, and entry angle of 120°. The previously dried composite material (laboratory dryer, 4 h at 80 °C) was placed in the rheometer cylinder and melted for 5 min. The composite was then extruded, and the pressure of the composite was determined before entering the measuring nozzle at a defined volumetric flow rate. The test was performed in the range of 11 s^−1^ to 690 s^−1^ of the uncorrected shear rate.

From the results obtained, corrected viscosity curves were determined using the Rabinowitsch correction. Viscosity curves were approximated using equally Ostwald de Waele, which allowed the melt flow exponent, *n*, to be determined. This parameter made it possible to assess the pseudoplastic properties of the melted composites. The procedure for calculating the rheological properties was described in earlier works [20,22,36,37,38].

The measurement was performed in at least 3 repetitions for each material. Results are presented as mean value with standard deviation.

#### 2.3.5. Plastographometric Analysis

The tests were performed using a FDO 234H plastographometer (Brabender, Duisburg, Germany). The material mixtures for testing were prepared in the same way as before the extrusion process. The dried wood flour was mixed with dry-blend PVC and immediately dosed into the plastograph meter using a 10 kg weight. The tests were conducted at a chamber temperature of 170 °C, a main rotor speed of 30 rpm, and a charge weight of 58 g. The measurement was carried out for 10 min. Based on the recorded changes in torque and temperature of the processed mixture, plastograms were plotted for further analysis. The torque and mass temperature after 0.5 min, 1 min, 2.5 min, 5 min, and 10 min were determined. The measurement was performed in at least 3 repetitions for each material. Results are presented as mean value with standard deviation.

## 3. Results

### 3.1. Tensile Properties

**Figure 1:**
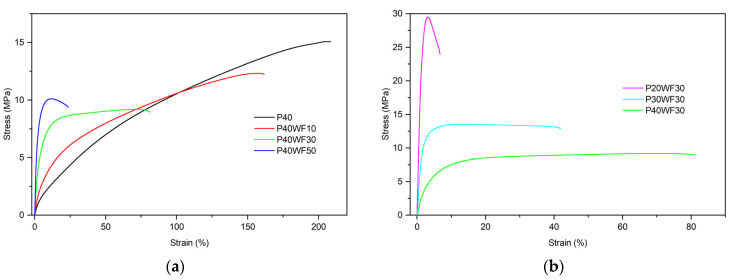
An exemplary strain–stress curve of plasticised PVC/wood composites: (**a**) depends on WF content and (**b**) depends on plasticiser content.

Figure 1 shows examples of tensile curves, while Table 3 presents characteristic values determined during the testing of the tensile properties of the tested materials.

The resulting composites have very different mechanical properties. The course of the tensile curves depends on both the proportion of filler and plasticiser. For plasticised poly(vinyl chloride) (P40, Figure 1a), the course of the relationship is characteristic of highly elastic materials: there is a gradual increase in stress with increasing strain, without a clearly defined c point [35,39]. The plasticiser present in the material significantly reduces intermolecular interactions by facilitating the orientation of polymer chains during progressive deformation [24,32,40]. The increase in filler content mainly increases the material’s stiffness, which is observed as a significantly higher increase in stress in the low strain range and an increase in the modulus of elasticity. Increasing the proportion of wood flour has a significant effect on reducing deformation at strength (Figure 1a). The wood filler significantly reduces the orientation of the polymer macromolecules [2,41,42]. Significantly, it was found that, with 50 phr of wood flour on the tensile curve, the yield stress could be re-established. It is possible that this is the result of some of the plasticiser being absorbed by the wood filler and reducing its efficiency. This can also be confirmed by analysing the variation in tensile curves determined for composites containing a fixed proportion of filler and a variable amount of plasticiser (Figure 1b). The incidence of yield stress was observed with a reduction in the proportion of plasticiser. The material’s strength decreases as the proportion of plasticiser and wood filler increases. It is worth noting that, for PVC matrix composites with 40 phr of plasticiser, composites containing 50 phr have significantly higher strengths compared to composites containing 30 phr of WF.

### 3.2. DMTA

**Figure 2:**
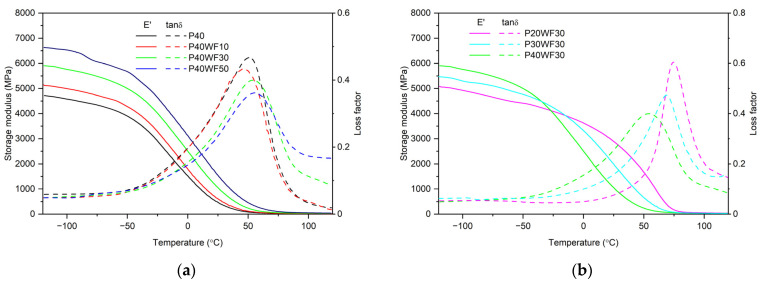
Exemplary DMTA thermograms of plasticised PVC/wood composites: (**a**) depends on WF content and (**b**) depends on plasticiser content.

In PVC technology, plasticisers are mainly used to lower the glass transition temperature, thus making it possible to obtain materials with high elasticity. An analysis of the dependence of changes in elastic properties over a wide temperature range is made possible by the DMTA analysis. DMTA thermograms of the obtained composites are shown in Figure 2. Characteristic values defining the extent of the glass transition region and storage modulus values are shown in Table 4.

From the analysis of the variation in *E*′ values, it can be concluded that the stiffness of the composites increases with the increasing filler content in the composite (Figure 2a), as noted from the mechanical-property tests. In addition, this relationship is maintained over the entire temperature range analysed.

A different relationship can be observed for composites containing a variable proportion of the plasticiser (Figure 2b). While at higher temperatures, an increase in the proportion of plasticiser increases the elasticity, at lower temperatures, the opposite relationship is observed. This is due to the freezing of the small-molecule plasticiser, which increases its viscosity and solidifies the whole material. At around −25 °C, the differences in stiffness between materials containing the same proportion of WF (Table 4, P20WF30, P30WF30, and P40WF30) and an extreme proportion of plasticiser are small. This is a characteristic phenomenon of plasticised poly(vinyl chloride) and largely depends on the properties of the plasticiser used [25,40,43]. For DINP, the freezing point is −43 °C. It is therefore worth knowing the specific properties in this respect when designing plasticised PVC-matrix composites for applications in a wide temperature range.

An important property of the materials produced is a significant reduction in the glass transition temperature. Increasing the proportion of plasticiser in the composite has a significant effect on lowering the glass transition temperature. This influence is evident across the entire range of the glass transition region, with the exception that increasing the proportion of wood flour reduces the effectiveness of the plasticiser. It is therefore possible that part of the plasticiser has been absorbed by the porous wood filler structure and thus cannot effectively reduce the intermolecular interactions in PVC. Such an inference is consistent with the considerations made during the analysis of mechanical properties.

In the case of the materials analysed, the determined values of the max tan*δ* temperature undoubtedly lie within the high-elasticity region of the materials produced, so binding them to the glass transition temperature would be incorrect. Nevertheless, the same effect of plasticiser and wood flour on the value analysed is observed. An increase in the proportion of plasticiser lowers and an increase in the proportion of filler raises the temperature at which the tan*δ* maximum occurs. At the same time, the value of the tan*δ* maximum decreases with increasing proportions of both plasticiser and wood flour. The tan*δ* value determines the energy damping and dissipation capacity due to viscoelastic interactions in the material [39]. An increase in the proportion of rigid filler particles at the expense of plasticised poly(vinyl chloride) reduces the dispersion and energy-dissipation capacity, which is associated with a reduction in damping properties at this temperature value. However, from a practical application of composites, it should be noted that, in the temperature range from about −50 °C to about 50 °C, the tan*δ* value is higher the higher the proportion of plasticiser in the composite. This is due to the lower glass transition temperature and the reduction in the temperature of onset of maximum tan*δ,* above which the value of the damping coefficient decreases. Thus, the plasticiser can enhance the damping capacity of WPCs over a wide range of service temperatures.

### 3.3. Water Absorption

When it comes to the manufacture of wood–polymer composites, an important aspect is to know their water-absorbing capacity. The determination of this characteristic gives information about the applicability of these composites under increased moisture conditions. It is also possible to indirectly assess the change in interphase interactions between the polymer and the wood particles, as this phenomenon is capillary in nature and largely depends on the space between the wood filler and the polymer matrix [44,45,46].

**Figure 3:**
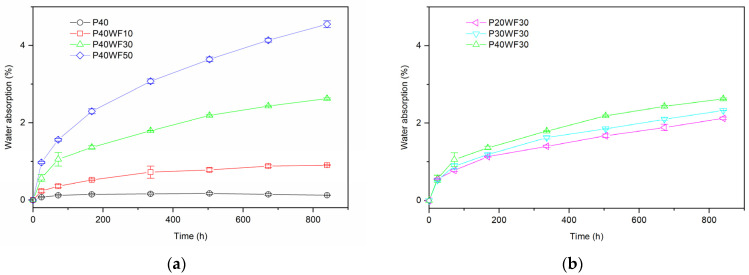
Water-absorption curves of plasticised PVC/wood composites: (**a**) depends on WF content and (**b**) depend on plasticiser content.

The course of the change in water absorption as a function of exposure time in water is shown in Figure 3.

The greatest increases in absorbency values occur at the onset of water exposure. With time, the kinetics of the change in water absorption and the rate of increase in sample weight decrease. This is due to the fact that the water absorption of WPCs, as well as polymers, follows Fick’s law [47,48]. Plasticised poly(vinyl chloride) is characterised by negligible water absorption. The water absorption after 840 h was 0.12% (Figure 3a). The wood particles, despite being surrounded by polymer, are able to absorb water. It is therefore clear that, as the proportion of wood flour in the composite increases, the absorption will increase significantly. In addition, the higher proportion of wood flour greatly facilitates the migration of water between the individual wood particles, which are in greater contact with each other. It was expected that, as a result of the use of plasticised poly(vinyl chloride) as a WPC polymer matrix, a significant increase in the water absorption of WPCs compared to composites with a different matrix would be observed. It was suspected that the flexible polymer matrix would not inhibit swelling of the wood particles, and this could consequently lead to significant water absorption. The absorbency value of the composites tested does not differ from the literature data for composites with a similar proportion of wood flour in a rigid PVC matrix [49]. Based on an analysis of water absorption for composites with the same proportion of wood flour but a varying proportion of plasticiser, a small effect of plasticiser on this property was shown (Figure 3b). For example, the water absorption of P20WF30 after 840 h of testing was 2.2%, while for P40WF30 composites, it was 2.6%, respectively. Summarising the water absorption analysis, it can be expected that wood–polymer composites with a plasticised PVC matrix will have the ability to be used in wet conditions.

### 3.4. Plastographometric Analysis

**Figure 4:**
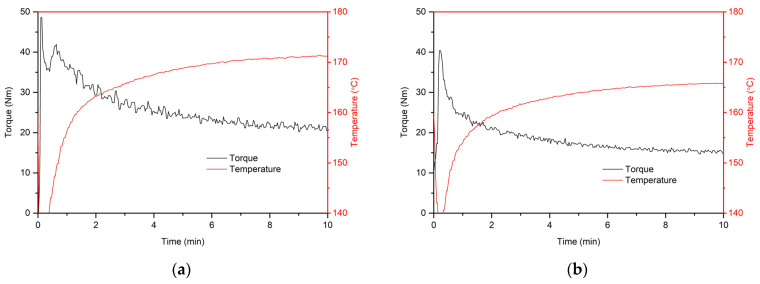
Exemplary plastograms of plasticised PVC/wood composites: (**a**) P20WF30 and (**b**) P40WF50.

**Figure 5:**
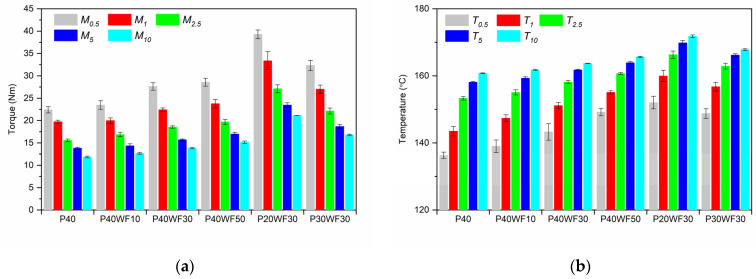
Characteristic values of the processed material for different processing times: (**a**) torque and (**b**) mass temperature.

An important application aspect of composites is to know more than just their performance properties. Their processing properties are extremely important, as they guarantee that these composites can be produced economically and correctly. In the case of the PVC-processing analysis, the assessment of the processability of the materials is assessed on the basis of plastograph tests, using torque rheometers [50,51,52]. The plastograms obtained during the test make it possible to estimate the changes occurring in melting during processing, e.g., by extrusion. Example plastograms determined for WPCs are shown in Figure 4, while the established characteristic torque and temperature values of the processed material for different processing times are summarised in Figure 5.

A majority of the samples tested exhibit the classic kneading process that is characteristic of plasticised poly(vinyl chloride), as shown in the plastogram of Figure 4b. That is, the gelation of the material takes place very quickly, as soon as the plastograph chamber is loaded. The characteristic torque maximum determined for rigid PVC is only found for the material P20WF30. The plastogram is shown in Figure 4a. The gelation time for this sample was 36 s, and the torque and mass temperature values at this point were 42 Nm and 147 °C, respectively.

The addition of wood flour and a reduction in the proportion of plasticiser in the PVC blends analysed had the effect of increasing the torque and temperature of the processed polymeric materials over the entire range of plastograph tests. These relationships are related to changes in rheological properties. An increase in filler content increases viscosity, while an increase in plasticiser content decreases viscosity. A broader analysis of the rheological properties is presented when discussing rheological tests. The increased temperature of materials with higher wood flour content and lower plasticiser content is related to the self-heating of the material due to the occurrence of a higher shear forces. It was not found that the addition of wood flour had a significant effect on facilitating the grinding of PVC grains, as is the case during the processing of unplasticised PVC [50,53]. In the case of plasticised PVC, the grains are pre-softened via the absorption of the plasticiser, and their gelling is much faster, and as a result, the supporting effect of the wood flour is imperceptible.

Significantly, no increase in torque at the end of the test, which could have been indicative of the onset of material degradation, was observed from the plastograph analysis. Thus, under the temperature conditions analysed, these materials show sufficient thermal stability for classical processing. In addition, by stabilising the torque values, it can be concluded that the wood flour does not increase the evaporation of the plasticiser during processing.

### 3.5. Rheological Properties

**Figure 6:**
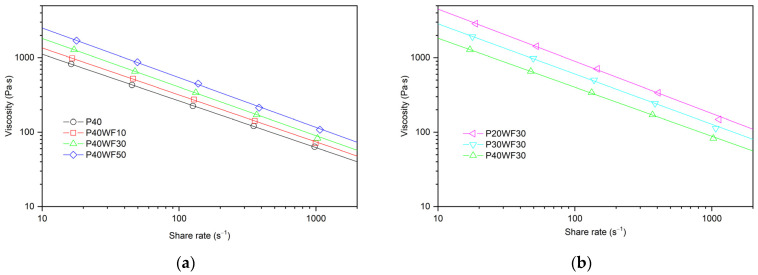
Corrected viscosity curves of plasticised PVC/wood composites (**a**) depend on WF content and (**b**) depend on plasticiser content.

Thermoplastic polymer composites in the plasticised state containing irregular filler particles, such as wood particles, are a complex rheological system to describe [54,55,56]. In addition, a plasticiser was introduced in the materials analysed, which influences a significant change in the interactions between the polymer chains, which manifests itself not only by changing the glass transition temperature but also by significantly altering the properties in the plasticised state. The corrected viscosity curves describing the basic rheological properties of the tested composites are shown in Figure 6, and the approximation data from the Ostwald de Waele law equation used are summarised in Table 5.

As the proportion of wood flour in the composite increases, the viscosity of the material increases significantly (Figure 6a), a result which coincides with basic rheological models describing the effect of fillers on the properties of polymer composites [55,56,57]. This relationship is maintained over the entire range of shear rates analysed, with the difference between the viscosity of the unfilled polymer and the composites decreasing as the shear rate increases. This applies to both the absolute difference in viscosity and the relative difference in viscosity, e.g., relative to the viscosity of the polymer without wood filler. This is also evidenced by the lower values of the flow exponent, *n*, which determines the pseudoplastic properties. The lower the value of *n*, the greater the shear dilution. At a shear rate of 20 s^−1^, the viscosity was 722 Pa s for P40, while the viscosity of P40W50 was 119% higher. In contrast, at a shear rate of 1000 s^−1^, the viscosity was 62 Pa s for P40 and only 87% higher for the P40W50 composite. Such relationships are characteristic of polymer–wood composites. This is due to the increased orientation of the filler in the direction of shear forces, so that the effect of the filler on viscosity at higher shear rates is lower [20,56,58,59].

The viscosity of the composites is also strongly influenced by the proportion of plasticiser. Increasing the amount of plasticiser significantly reduces the viscosity of the composite (Figure 6b). Plasticisers interact with polymer macromolecules in the molten state, reduce intermolecular interactions, and can also act as a lubricant by reducing internal resistance. In a WPC system, it is possible that this applies not only to polymer–polymer interactions but also to polymer–filler and filler–filler interactions. As the proportion of plasticiser increased, a reduction in the melt exponent value was also found. Thus, analogous to the analysis of the effect of the filler, as the proportion of shear rate increases, the difference in relative viscosity related to one of the systems as the proportion of plasticiser increases will decrease. In this case, one could speculate that the plasticiser should facilitate the orientation of the macromolecules, thereby enhancing the pseudoplastic properties. However, this modifier is a low-molecular-weight substance with markedly different rheological properties; thus, its significant contribution further affects the rheological properties of the modified material. In the case of composites with a lower plasticiser content, higher shear stresses occur. This can lead to significant thermal effects associated with energy dissipation, as demonstrated by plastographometric studies. This phenomenon may also have the effect of further reducing the viscosity of the composite as the shear rate increases.

## 4. Conclusions

On the basis of the work carried out, it was shown that it is possible to produce polymer–wood composites based on a plasticised poly(vinyl chloride) matrix characterised by a wide range of both functional and processing properties.

As the proportion of wood flour increases, there is a significant increase in stiffness, water absorption, and viscosity, along with a decrease in maximum strain and strength. On the other hand, an increase in the proportion of plasticiser has a significant effect on increasing elasticity and elongation at break and decreasing the glass transition temperature and viscosity. Importantly, for application purposes, despite the use of a flexible polymer matrix, there was no significant effect of the plasticiser on the water absorption phenomenon.

It should be emphasised that the significant proportion of wood flour, which is likely to act as a plasticiser sorbent, reduces the effectiveness of the plasticiser used, and this fact should be taken into account when designing the composition of PVC mixtures.

## Figures and Tables

**Table 1:** List of the composition of composites and the composite markings used.

Material	Contribution of Components to the Material, phr				
	Dry Blend				Wood Flour
	PVC	DINP	Patstab 2301	Naftolube FTP	
P40	100	40	4	1	0
P40WF10	100	40	4	1	10
P40WF30	100	40	4	1	30
P40WF50	100	40	4	1	50
P20WF30	100	20	4	1	30
P30WF30	100	30	4	1	30

**Table 2:** Temperature values of individual heating zones during processing.

Process	Processing Temperature Setting, °C										
Extrusion	z1	z2	z3	z4	z5	z6	z7	z8	z9	Connector	Head
	90	100	120	130	140	145	155	170	170	165	155
Injection moulding	z1		z2		z3		z4			Injection mould	
	90		120		170		170			18	

**Table 3:** Characteristic values of tensile properties’ analysis.

Material	Tensile Modulus, MPa	Tensile Strength, MPa	Strain at Strength, %
P40	18 (1)	15.0 (0.2)	205 (5)
P40WF10	34 (1)	12.3 (0.2)	153 (2)
P40WF30	112 (3)	9.1 (0.1)	71 (2)
P40WF50	274 (8)	10.2 (0.1)	12 (0)
P20WF30	1760 (40)	29.5 (0.6)	3 (0)
P30WF30	463 (9)	13.5 (0.1)	14 (1)

**Table 4:** Characteristic values of DMTA analysis.

Material	*T_g_*, *E*′, °C			*E*′, MPa			tan*δ*_max_, °C
	Onset	Inflection	Offset	−25 °C	0 °C	25 °C	
P40	−46.3 (0.3)	−14.9 (2.8)	25.7 (0.4)	2925 (25)	1538 (21)	487 (18)	49 (2)
P40WF10	−42.5 (0.4)	−4.2 (1.3)	26.6 (1.9)	3346 (84)	1763 (46)	643 (40)	45.6 (3.0)
P40WF30	−40.3 (0.2)	0 (1.1)	36.6 (0.4)	3911 (319)	2429 (209)	1034 (109)	53.1 (0.2)
P40WF50	−36.6 (2.2)	2.8 (3.7)	42.5 (0.9)	4335 (420)	2909 (315)	1418 (181)	5.9 (0.6)
P20WF30	21.2 (0.9)	59.8 (0.6)	73.3 (1.1)	4092 (121)	3626 (87)	2893 (72)	74.7 (0.8)
P30WF30	−20.5 (0.5)	26.4 (0.9)	57.2 (0.7)	4242 (142)	3334 (131)	1994 (94)	69.4 (0.8)

**Table 5:** Ostwald de Waele law equation parameter.

Material	*n*,	*k*, Pa·s*^n^*	R^2^
P40	0.373 (0.004)	4732 (64)	1
P40WF10	0.369 (0.006)	5793 (127)	0.9997
P40WF30	0.341 (0.006)	8337 (201)	0.9997
P40WF50	0.333 (0.008)	11,688 (356)	0.9996
P20WF30	0.299 (0.011)	22,652 (1043)	0.9989
P30WF30	0.347 (0.004)	8189 (113)	0.9999

## Data Availability

The raw data supporting the conclusions of this article will be made available by the authors on request.
